# Determinants of overweight and obesity among Bangladeshi diabetic women of reproductive age

**DOI:** 10.1186/1756-0500-7-513

**Published:** 2014-08-11

**Authors:** Jesmin Akter, Mohammad Shahjahan, Sharmin Hossain, Hasina Akhter Chowdhury, Kazi Rumana Ahmed, Kaniz Fatema, Begum Rowshan Ara, Liaquat Ali

**Affiliations:** Department of Reproductive and Child Health, Bangladesh University of Health Sciences (BUHS), Darus Salam, Mirpur, Dhaka 1216 Bangladesh; Department of Public Health, Daffodil International University (DIU), Dhaka, Bangladesh; Department of Health Promotion and Heath Education, BUHS, Darus Salam, Mirpur, Dhaka 1216 Bangladesh; Department of Biostatistics, BUHS, Darus Salam, Mirpur, Dhaka, 1216 Bangladesh; School of Public Health and Community Medicine, University of New South Wales, Sydney, Australia; Department of Biochemistry and Cell Biology, BUHS, Dhaka, 1216 Bangladesh

**Keywords:** Overweight and obesity, Diabetes, Reproductive age, Bangladesh

## Abstract

**Background:**

Several reproductive disorders with overweight and obesity are now known to be associated with insulin resistance. The study was aimed to assess the proportion and determinants of overweight and obesity among diabetic women of reproductive age.

**Methods:**

This cross-sectional analytic study was conducted among 888 diabetic women of reproductive age attending the out-patient department (OPD) of the central hospital of the Diabetic Association. Body Mass Index (BMI) was used to assess the general obesity. Waist Circumference (WC), Waist-Hip Ratio (WHR) and Waist-Height Ratio (WHtR) were used to assess central obesity.

**Results:**

The overall prevalence of overweight was 22% (95% CI 19-24) and that of obesity was 48% (95% CI 45-51). Prevalence of central obesity by waist circumference was 77%, by waist-hip ratio was 99.9% and by waist-height ratio was 89%. Overweight and obesity were higher in the age group of 45-49 years (49%) and 35-44 years (24%) respectively. On Pearson’s correlation analysis, BMI and WC were significantly correlated with age (r = 0.135, p = 0.001; r = 0.162, p = 0.001) and income (r = 0.151, p = 0.001; r = 0.087, p = 0.009) respectively. WHR was also correlated with income (r = 0.094, p = 0.005). Moreover, WHtR was significantly correlated with age (r =  0.139, p = 0.001), income (r = 0.069, p = 0.04) and duration of diabetes (r = 0.073, p = 0.03).

On binary logistic regression analysis, BMI was significantly associated with age, income and management of diabetes by Oral Hypoglycemic Agent (OHA) (p < 0.05). WC was significantly associated with age, income and management of diabetes by OHA and insulin (p < 0.05). Where, WHR was significantly associated only with duration of diabetes (p < 0.05). WHtR was significantly associated with age (p < 0.05), management of diabetes by OHA (p < 0.05) and insulin (p < 0.05) in this analysis.

**Conclusions:**

A high prevalence of both overweight and obesity exists in diabetic women of reproductive age in Bangladesh and it seems to be associated with increasing age, income, duration of diabetes, and use of oral hypoglycemic agents.

## Background

Obesity is increasing at an alarming rate throughout the world and has become a global problem [1]. The World Health Organization (WHO) has declared overweight as one of the top of 10 health risks in the world and one of the top five in developed nations [2]. According to recent estimates, there are more than one billion overweight people worldwide, and some 250 million of these are estimated to be clinically obese [3], equivalent to 7% of the world adult population. Once considered a problem related to affluence, obesity is now fast growing in many developing countries and in poor neighborhoods of the developed countries [4, 5]. Nowadays, there is an increasing trend of overweight and obesity is found to be common in Bangladesh [6]. Incident of rapid demographic transition, sustainable economic development, rapid urbanization, and changing lifestyle and dietary patterns are some of the important causal factors for the emergence of overweight and obesity in developed and developing countries [7].

The etiology of obesity is multi factorial. Poor diet and physical inactivity cause overweight and obesity. This imbalance between food intake and energy expenditure is determined, in large part, by the socioeconomic context. Although obesity is affected by interaction between multiple genes and the environment, the genetic pool is not changing rapidly; it is the environmental and social context that has changed and caused the epidemic [8].

Furthermore, this chronic condition has been linked to the development of diabetes and cardiovascular disease, endometrial, colon, postmenopausal breast, and other cancers; and certain musculoskeletal disorders, such as knee osteoarthritis later in life [9]. It is also a critical public health problem for women of reproductive age. Obesity has been associated with both short- and long-term health effects for women as well as for their offspring’s. Existing research supports a link between obesity and conditions that impair a woman’s ability to conceive and increase her risk for an adverse pregnancy outcome [10, 11].

There is a large literature demonstrating that women who are overweight are at greater risk of developing pregnancy complications and problems associated with labor and delivery. Finally, obese women are more at risk of postpartum complications. Taken all complications, maternal mortality and morbidity is significantly elevated for obese women [12–14]. Maternal obesity is also related with health hazards for the fetus and the newborn. Obese women are more likely to give birth to a fetus with congenital anomalies overweight and to have infants who are exposed to a significantly higher perinatal morbidity rate [15]. The effects of maternal obesity do not stop at birth - research has shown that babies born to obese mothers are at greater risk of later developing diabetes, cardiovascular disease and obesity themselves [16].

In most regions of the world, overweight now exceeds underweight among women of reproductive age [4]. Obesity is the tip of the iceberg of a cluster of cardiovascular disease (CVD) risk factors, including hypertension and dyslipidemia. The natural consequence will be an epidemic of cardiovascular complications among diabetic patients, such as coronary heart disease and stroke as well as microvascular complications. Obesity also increases the risk of several reproductive disorders, negatively affecting normal natural function and fertility [17].

In Bangladesh recently the overweight and obesity among the women of reproductive age is increasing [18]. From this point of view, an intensive research on the dynamics of obesity is needed to understand this upcoming health issue and formulate effective programs to enhance the quality of life of the people. However, due to paucity of data, understanding of the causes and consequences of this rapidly growing public health threat remains poor. Therefore, this study is an attempt to understand the dynamics of obesity among women in Bangladesh perspective. The objective of the present study was to assess the proportion and determinants of overweight and obesity among diabetic women of reproductive age attending a tertiary hospital in Bangladesh.

## Methods

This cross-sectional analytic study was conducted among 888 diabetic women of reproductive age attending the out-patient department (OPD) of Bangladesh Institute of Research & Rehabilitation on Diabetes, Endocrine and Metabolic Disorders (BIRDEM), Dhaka. BIRDEM is a central tertiary hospital of the Diabetic Association of Bangladesh. Diabetic patients from all over the country including referred patients from allied associations are destined here. Being a charity the hospital attracts patients with different demographic and socioeconomic background from all over the country. Thus, data generated from this hospital may be considered as a fair reflection of general diabetic population of the country.

The duration of the study extended for 9 months during May 2011 to January 2012. Data were collected using a semi-structured questionnaire by face to face interview. Purposive sampling technique was used during data collection. No such data were available for Bangladeshi reproductive diabetic population so far reviewed. The prevalence of diabetic women were found from the summarized data of Knowledge Attitude and Practice (KAP) study for World Diabetes Foundation (WDF) 05–131 and considered here for estimating the study sample [19]. Patients were asked to control their diabetes as - lifestyle management (diet and exercise), insulin and OHA or Insulin/OHA combination. 24 hours dietary recall method was used to calculate energy intake of the study subjects. Duration of diabetes was 1–13 years. The socio-economic classification in this study was made according to 2006 Gross National Income (GNI) per capita and using the calculation of World Bank (WB) [20] (The groups were: low-income $75.41 or less (BDT ≤ 5360), lower middle-income $75.5 - $299.58 (BDT 5361–21270), upper middle-income $299.68 - $926.25 (BDT 21271–65761) and high-income $926.33 or more (BDT ≥ 65762)).

The weighing machine (Model number: RGZ-160, China) was used for measuring the body weight. Before using the machine every time the balance was rectified. Body weight was measured in bare foot having light clothes (nearest 0.1 kg). Participants were informed to come for measurement in empty stomach and empty bladder. Height was measured in bare foot in standing position with a standard scale to nearest 0.1 cm. At the time of measuring height, the participants were instructed to stand straight with eye looking forward, head positioned horizontally, feet together and knee straight. The participants were asked to stand and hold their own gown. Then examiner found an imaginary point crossing the horizontal line of iliac crest and the midaxillary line of the body. Then the participants were requested to lower slightly the pants and underclothing to palpate directly on the hip area for the iliac crest. Then waist circumference was measured by a measuring tape, using it parallel on those points. The measurements were made at minimal respiration to the nearest 0.1 cm. Hip circumference was measured at the greatest protrusion of the buttocks just below the iliac crest. The measurement was taken in centimeters.

BMI [WHO Guideline for South East Asian (SEA) population] was used to assess the general obesity. Waist Circumference (WC, IDF criteria for SEA population), Waist-Hip Ratio (WHR, IDF criteria for SEA population) and Waist-Height Ratio (WHtR, IDF criteria for SEA population) were used to assess central obesity [21–24]. Cut off points of overweight and obesity for both sexes were ≥23–24.9 kg/m^2^ and ≥25 kg/m^2^ respectively. Cut off points of waist circumference for men and women were ≥90 and ≥80 cm, WHR for men ≥0.90 and women ≥0.80 and WHtR for both sexes were ≥0.50 respectively.

All data were expressed as the mean ± SD and percentage. To assess the significant association of quantitative data Pearson’s correlation coefficient with p-values was calculated to explore the association between two variables. Binary logistic regression were performed to quantify the individual effect of predictor variables and to adjust for potential confounding factors. The statistical tests were considered significant at a level ≤5% (≤0.05). Data were presented by tables and graphs. All the statistical analysis was performed using SPSS 17 software.

## Results

### Patient’s characteristics

Age of the study participants ranged between 15 and 49 years with a mean (±SD) age of 39 ± 7 years. About half of the participants were in the age group of 35–44 years (43.7%). The higher proportion of the participants was housewives (94.9%). Majority of participants (43%) were educated up to primary level and 58% of the participant’s belonged from lower middle class (monthly household income between 5361 and 21270 taka) (Table 1).

**Table 1:** **Demographic and socio-economic characteristics of the study subjects (n = 888)**

Variables	Number	Percentage
*Age (Mean ± SD)*	39 ± 7	
15-24 yrs	35	3.9
25-34 yrs	181	20.4
35-44 yrs	388	43.7
45-49 yrs	284	32.0
*Occupation*		
Service	27	3.0
Housewife	843	94.9
Others	18	2.1
*Education*		
Illiterate	256	29
Primary	378	43
Secondary up to HSC	204	23
Graduate and above	50	5
*Socio-Economic status*		
Low income (<5360)	152	17
Lower middle income (5361–21270)	511	58
Upper middle income (21271–65761)	195	22
High income (>65762)	30	3
*Duration of diabetes (in years) (Mean ± SD)*	4.9 ± 4.3	
<5 years	616	67
6-10 years	205	22
11+ years	99	11
*Management of diabetes*		
Diet/Exercise	367	41.3
OHA	198	22.3
Insulin	231	26.0
Combination (Insulin + OHA)	92	10.4

Results are expressed as number (percentage) and mean ± SD.

### Prevalence of overweight and obesity

The overall prevalence of overweight was 22% (95% CI 19–24) and that of obesity was 48% (95% CI 45–51) (Table 2). Prevalence of central obesity by waist circumference was 77%, by waist-hip ratio was 99.9% and by WHtR 89% (Table 3). Overweight and obesity were higher in the age group of 45–49 years (49%) and 35–44 years (24%) respectively.

**Table 2:** **Prevalence of overweight and obesity according to BMI (n = 888)**

Variables	Prevalence (%)	95% CI
Overweight *(23–24.9)*	22	19-24
Obese *(>25)*	48	45-51

Results are expressed as number, percentage and Confidence Interval (CI).

**Table 3:** **Anthropometric indices of the study subjects (n = 888)**

Mean ± SD variables	Number	Percentage
BMI (Mean ± SD)	25 ± 4	
*Underweight (<18.5)*	35	4
*Normal (18.5-22.9)*	234	26
*Overweight (23–24.9)*	192	22
*Obese (>25)*	427	48
WC (in cm) (Mean ± SD)	88 ± 11.2	
*Normal (<80)*	204	23
*Health Risk (>80 & above)*	684	77
WHR (Mean ± SD)	0.97 ± 0.08	
*Normal (<0.80)*	1	0.1
*Health Risk (>0.80 & above)*	887	99.9
WHtR (Mean ± SD)	0.58 ± 0.08	
*Normal (<0.50)*	5	11
*Health Risk (>0.50 & above)*	883	89

Results are expressed as number (percentage) and mean *±* SD, BMI = Body mass index, WC = Waist circumference, WHR = Waist to hip ratio, WHtR = Waist to height ratio.

### Determinants of overweight and obesity

The Table 4 demonstrates correlation coefficient generated through Pearson correlation. Age and income were significantly correlated with BMI (r = 0.135, p = 0.001; r = 0.151, p = 0.001), WC (r = 0.162, p = 0.001; r = 0.087, p = 0.009) and WHtR (r = 0.139, p = 0.001; r = 0.069, p = 0.04) respectively. Where, only income was correlated with WHR (r = 0.094, p = 0.005). On the other hand, duration of diabetes was significantly correlated only with WHtR (r = 0.073, p = 0.03).

**Table 4:** **Association of anthropometric indices with other variables**

Variables	BMI	WC	WHR	WHtR
	r/p	r/p	r/p	r/p
Age	.135/.001	.162/.001	.051/.126	.139/.001
Income	.151/.001	.087/.009	.094/.005	.069/.04
Duration of diabetes	-.015/.665	.057/.088	.051/.131	.073/.03
Energy intake	.001/.998	.017/.619	-.035/.296	.000/.976

The level of significance at p < 0.05; r = correlation coefficient.

On binary logistic regression analysis, BMI was significantly associated with age (p < 0.05), income (p < 0.05) and management of diabetes by OHA (p < 0.05). On the other hand, WC was significantly associated with age (p < 0.001), income (p < 0.05), management of diabetes by OHA (p < 0.05) and insulin (p < 0.05). WHR was significantly associated only with duration of diabetes (p < 0.05) and WHtR was significantly associated with age (p < 0.05), management of diabetes by OHA (p < 0.05) and insulin (p < 0.05) (Table 5).

**Table 5:** **Logistic regression coefficient and odds ratio (95% CI) of determining variables considering different variable**

Variables	BMI		WC		WHR		WHtR	
	P-value	OR (95% CI)	P-value	OR (95% CI)	P-value	OR (95% CI)	P-value	OR (95% CI)
Age	0.001	1.04 (1.02-1.06)	0.001	1.03 (1.01-0.06)	0.706	0.98 (0.93-1.04)	0.003	1.04 (1.02-1.07)
Income	0.016	1.00 (1.0-1.0)	0.030	1.00 (1.0-1.0)	0.865	1.00 (1.0-1.0)	0.249	1.00 (1.0-1.0)
Duration of diabetes	0.513	0.98 (0.95-1.02)	0.438	1.01 (0.97-1.05)	0.013*	1.25 (1.04-1.49)	0.506	1.02 (0.9-1.1)
Management of diabetes								
Diet/exercise*	Reference		Reference		Reference		Reference	
*OHA*	0.047	1.88 (1.2-2.8)	0.001	4.16 (2.4-7.1)	0.441	1.59 (0.48-5.2)	0.003	2.64 (1.4-5.0)
*Insulin*	0.567	1.11 (0.77-1.5)	0.013	1.65 (1.1-2.4)	0.495	0.70 (0.25-1.9)	0.001	2.53 (1.43-4.4)
*Combination*	0.611	1.14 (0.68-1.9)	0.583	1.16 (0.68-1.9)	0.943	0.94 (0.19-4.5)	0.286	1.47 (0.72-2.9)
Exercise habit								
No*	Reference		Reference		Reference		Reference	
*Yes*	0.466	1.15 (0.78-1.6)	0.937	1.00 (0.65-1.5)	0.230	2.49 (0.56-11.1)	0.129	1.50 (0.8-2.5)
Energy intake	0.448	1.00 (1.0-1.0)	0.488	1.00 (1.0-1.0)	0.437	1.00 (0.99-1.0)	0.337	1.00 (0.9-1.0)

*: Reference group, β for standardized regression coefficient, BMI, WC, WHR and WHtR were taken as dependent variables whereas others taken as independent variables. Significant at p-value <0.05 levels, CI = Confidence Interval, OR = odds ratio, OHA = Oral Hypoglycemic Agent.

## Discussion

Noticeably, overweight or obesity is associated with high mortality, disability, and a poor quality of life [25]. Research showed that current rate of overweight and obesity are already unacceptably high among women [26]. This is of considerable concern for number of reasons. This is the risk factor of diabetes mellitus, hypertension, dyslipidemia, CHD, depression and various type of cancer. This increasing trend of overweight and obesity, when combined with the continued trend toward globalization, will serve to seriously escalate the population level of obesity. This study showed that 22% and 48% of the Bangladeshi diabetic women of reproductive age are overweight and obese respectively according to Asian specific BMI cut-off value. Thus the results of the present study show that overweight and obesity are major public health problems for reproductive age women in Bangladesh. No study was conducted on Bangladeshi diabetic women of reproductive age for measuring overweight and obesity previously. In Saudi Arab, El Hazmi et al. [27] conducted a study, where 29.66% and 39.27% diabetic women were found overweight and obese respectively by using WHO standard BMI cut off value. Our study showed more prevalence of overweight and obesity in our population. Probably the cut-off value of BMI and ethnicity are the leading cause of difference of this result. Khan et al. [25] conducted a study among ever-married non-pregnant urban women in Bangladesh. They had found 15.7% and 3.9% of participants were overweight and obese respectively. The probable reason of difference may be due to the study population and cut-off values. But now this situation has changed dramatically due to rapid demographic transition from traditional lifestyle to modern culture. The prevalence of obesity has reached on its peak, near about 100%, among both Saudi Arabian and Bangladeshi women. The current study found 77% respondent had excess waist circumference and its mean was 88.0 ± 11.2 cm. Anisur et al. [28] found 23% urban women at Dhaka had excess waist circumference, which is much lower than our study findings. Surprisingly, almost hundred percent (99.9%) diabetic women had excess cut-off value of waist to hip ratio and they fall in the higher risk group of obesity related disorders in this study. Most of the respondents had abnormal waist to height ratio (89%). Women had more body fat composition than men and the values are probably high due to the inclusion of the diabetic population in this study.

The highest prevalence of overweight and obesity has been reported in women aged 35–49 age groups and correlation curve show that BMI, waist circumference and waist-height ratio are increased with advancement of age. This result was similar with Sotoudeh et al. [29] also found that obesity is increased with age. The higher income group people have a sedentary lifestyle and it can induce obesity. The present study shows that increasing household income is associated with anthropometric markers (BMI, WC, WHR and WHtR). Duration of diabetes is also positively associated with waist to height ratio. In present study, by multivariate analysis, age, income levels and duration of diabetes are found to independently correlate with overweight and central obesity. Using of oral hypoglycemic drug for treatment of diabetes is found to create abnormal anthropometric risk markers among diabetic reproductive aged women. OHA users had near about 4 times higher chance of development of central obesity and 2 times higher chance of over weight than the lifestyle modification adopting groups. Drug trials showed that some OHA can induce obesity [30] and which support our research findings. As the samples were drawn from one hospital, several factors may have impact on the results as to limit its generalizability. However, as mentioned earlier BIRDEM is an institute of the Diabetic Association of Bangladesh (DAB), which functions as a referral hospital for whole of Bangladesh through DAB’s nationwide referral centers.

## Conclusions

The prevalence of overweight and obesity among women of reproductive age seems to be a critical public health concern. A high prevalence of both overweight and obesity exists in diabetic women of reproductive age in Bangladesh and it seems to be associated with increasing age, income, duration of diabetes, management of diabetes especially use of oral hypoglycemic agent. Our findings confirm and reinforce the need for further attention to the health and wellbeing of women of reproductive age to prevent the epidemic of overweight and obesity.

### Ethical consideration

This study was approved by the Ethical Review Committee of Diabetic Association of Bangladesh (BADAS). Written informed consent was obtained from the all participants. Ethics has been respected throughout the whole study period.
